# *In Vitro* Gut Modeling as a Tool for Adaptive Evolutionary Engineering of *Lactiplantibacillus plantarum*

**DOI:** 10.1128/mSystems.01085-20

**Published:** 2021-04-13

**Authors:** Julia Isenring, Annelies Geirnaert, Alex R. Hall, Christoph Jans, Christophe Lacroix, Marc J. A. Stevens

**Affiliations:** a Laboratory of Food Biotechnology, Institute of Food, Nutrition and Health, ETH Zurich, Zürich, Switzerland; b Institute of Integrative Biology, ETH Zurich, Zürich, Switzerland; c Institute for Food Hygiene and Safety, University of Zurich, Zürich, Switzerland; Pontificia Universidad Catolica de Chile

**Keywords:** adaptive evolutionary engineering, colonic microbiota, *in vitro* gut modeling, *Lactiplantibacillus plantarum*

## Abstract

Research and marketing of probiotics demand holistic strain improvement considering both the biotic and abiotic gut environment. Here, we aim to establish the continuous *in vitro* colonic fermentation model PolyFermS as a tool for adaptive evolutionary engineering. Immobilized fecal microbiota from adult donors were steadily cultivated up to 72 days in PolyFermS reactors, providing a long-term compositional and functional stable ecosystem akin to the donor’s gut. Inoculation of the gut microbiota with immobilized or planktonic Lactiplantibacillus plantarum NZ3400, a derivative of the probiotic model strain WCFS1, led to successful colonization. Whole-genome sequencing of 45 recovered strains revealed mutations in 16 genes involved in signaling, metabolism, transport, and cell surface. Remarkably, mutations in LP_RS14990, LP_RS15205, and intergenic region LP_RS05100<LP_RS05095 were found in recovered strains from different adaptation experiments. Combined addition of the reference strain NZ3400 and each of those mutants to the gut microbiota resulted in increased abundance of the corresponding mutant in PolyFermS microbiota after 10 days, showing the beneficial nature of these mutations. Our data show that the PolyFermS system is a suitable technology to generate adapted mutants for colonization under colonic conditions. Analysis thereof will provide knowledge about factors involved in gut microbiota colonization and persistence.

**IMPORTANCE** Improvement of bacterial strains in regard to specific abiotic environmental factors is broadly used to enhance strain characteristics for processing and product quality. However, there is currently no multidimensional probiotic strain improvement approach for both abiotic and biotic factors of a colon microbiota. The continuous PolyFermS fermentation model allows stable and reproducible continuous cultivation of colonic microbiota and provides conditions akin to the host gut with high control and easy sampling. This study investigated the suitability of PolyFermS for adaptive evolutionary engineering of a probiotic model organism for lactobacilli, *Lactiplantibacillus plantarum*, to an adult human colonic microbiota. The application of PolyFermS controlled gut microbiota environment led to adaptive evolution of *L. plantarum* strains for enhanced gut colonization characteristics. This novel tool for strain improvement can be used to reveal relevant factors involved in gut microbiota colonization and develop adapted probiotic strains with improved functionality in the gut.

## INTRODUCTION

Microorganisms play a pivotal role in pharmaceutical, biotechnological, and food industries. The last depends heavily on microorganisms for starter cultures, biopreservation agents, and flavor producers (1–3). Moreover, since the 1990s, there has been an increase in the production of probiotics, which are “live microorganisms that, when administered in adequate amounts, confer a health benefit on the host” (4). Strain improvement of probiotic bacteria is of major importance to meet consumer demands for functional foods and enhance competitiveness of probiotic strains. However, it demands a multidimensional approach since biotic and abiotic factors are involved.

A promising solution for strain improvement is evolutionary engineering, which steers microbial evolution by exerting selective pressure (5–7). Desired mutants can be selected based on, e.g., growth rate, increased survival, or retention time. This method is feasible with bacteria because short generation times and large population sizes facilitate rapid emergence and selective sweeps of mutants (8–11). It is a well-established approach to improve targeted strain characteristics like the acidification rate of Lactococcus lactis (12), growth of Escherichia coli (13), or enhanced succinate production in *Actinobacillus* and *Mannheimia* (10, 14, 15). Nonetheless, the potential of evolutionary engineering as multidimensional engineering within microbial consortia is not well established yet. Previously, the residence time of Lactiplantibacillus plantarum in the murine digestive tract was increased after repetitive administration of the longest-persisting *L. plantarum* (16). However, *in vivo* models like mice have societal, ethical, and monetary restrictions and might therefore be replaced by *in vitro* models. Furthermore, gastrointestinal physiology and gut species composition of mice are different from humans, possibly limiting the translation (17, 18).

Continuous fermentation models are best suited for *in vitro* cultivation of gut microbiota in conditions akin to the gut (19, 20). Different PolyFermS models were successfully developed for cultivating colonic microbiota of humans of different ages and conditions and swine, murine, and chicken cecum microbiota (21–25). The continuous PolyFermS model allows testing several treatments in parallel in second-stage treatment reactors (TRs) seeded with the same gut microbiota produced in the inoculum reactor (IR) containing immobilized microbiota (21). Gut microbiota immobilization in polysaccharide gel beads leads to the maintenance of high cell density, long-term stability due to prevention of cell washout, and diversity of the simulated gut microbiota (23, 26, 27). It moreover creates a sessile bacterial fraction on the gel beads and a planktonic fraction resulting from the growth and release of sessile bacteria and further growth of planktonic cells in the bulk medium (27, 28). This mimics the gastrointestinal environment consisting of free and biofilm- or mucus-associated bacteria (29, 30). The PolyFermS colonic fermentation model enables operation up to several months in a highly controllable environment with multiple parameters to operate on, which is needed for evolutionary adaptation (26). We therefore hypothesized that the PolyFermS model can provide a long-term stable gut microbiota akin to the human adult colon that allows for adaptive evolution of an exogenous single strain.

In this study, we investigated the PolyFermS fermentation model as a novel tool for strain improvement via adaptive evolutionary engineering, using *L. plantarum* as a model strain. *L. plantarum* originates from fermented foods (31, 32) and is detected at low levels in approximately half of healthy human gut microbiota (33). *L. plantarum* WCFS1 is a well-characterized model strain for transient probiotic lactobacilli (34, 35). A WCFS1 derivative harboring a chloramphenicol (CM) resistance gene for tracking was cultivated in PolyFermS reactors inoculated with immobilized adult fecal microbiota for at least 100 generations. Engineered strains were phenotypically and genotypically characterized and tested for improved colonization in the PolyFermS model.

## RESULTS

### The PolyFermS model allows stable cultivation of adult gut microbiota.

**FIG 1:**
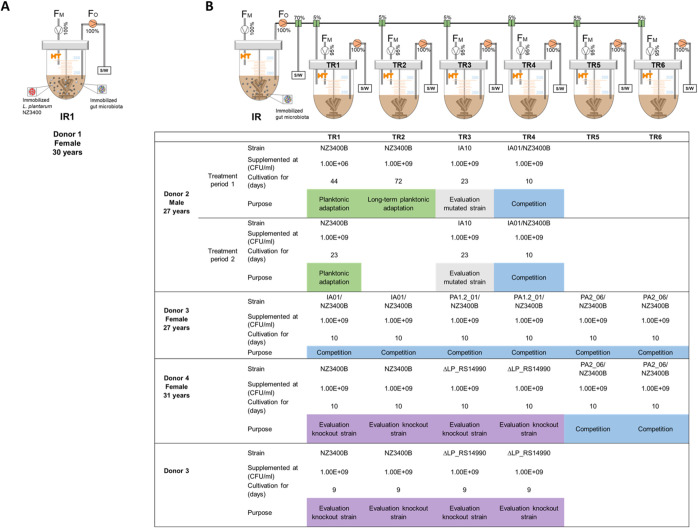
PolyFermS setup of immobilized and planktonic adaptation and further experiments in *in vitro* human adult gut microbiota. (A) Immobilized *L. plantarum* NZ3400 was added to immobilized fecal gut microbiota in IR1 from donor 1 during a single-stage fermentation. (B) Immobilized fecal microbiota of donors 2 to 4 were cultivated in the inoculum reactor (IR), which was used to inoculate second-stage reactors (TRs) that were supplemented with *L. plantarum* after stabilization. NZ3400, reference strain; NZ3400B, new stock from single-colony isolate of NZ3400; IA10, IA01, PA1.2_01, and PA2_06, recovered *L. plantarum* mutants; *L. plantarum* ΔLP_RS14990, LP_RS14990 gene deletion strain; F_M_, inflow MacFarlane medium; F_O_, reactor outflow; S/W, sampling/waste.

The PolyFermS model operated to mimic the adult proximal colon was used to provide a gut microbiota environment for evolutionary adaptation of *L. plantarum* NZ3400. Adaptation of immobilized *L. plantarum* was performed in IR1, and adaptation of planktonic *L. plantarum* was tested in TRs continuously inoculated with IR2 microbiota (Fig. 1). Metabolic stability of IR1 (see Fig. S2A in the supplemental material) was achieved after 1 week with main short-chain fatty acids (SCFAs) acetate, propionate, and butyrate at 73 ± 7, 21 ± 4, and 19 ± 3 mM over a 2-month fermentation, respectively. IR1 microbiota was dominated by *Firmicutes* and *Bacteroidetes* accounting for 44% ± 5% and 47% ± 2% of the total population during days 44 to 46, respectively; 48% ± 7% and 41% ± 2% during days 57 to 59, respectively; and 52% ± 4% and 36% ± 4% during days 61 to 65, respectively (see Fig. S3A in the supplemental material). This stability was also observed on a family level (see Fig. S3B in the supplemental material). IR2 microbiota reached metabolic stability (see Fig. S2B in the supplemental material) after 1 week with the main SCFAs acetate, propionate, and butyrate at 84 ± 5, 32 ± 8, and 23 ± 4 mM, respectively, during 2 months of culture. IR2 had a different microbiota composition from IR1 (see Fig. S3C and D in the supplemental material). *Bacteroidetes* dominated the gut microbiota compared to *Firmicutes* with 63% ± 1% and 36% ± 1% during days 24 to 26, respectively; 68% ± 4% and 30% ± 4% during days 41 to 43, respectively; and 66% ± 1% and 30% ± 1% during days 89 to 91, respectively (see Fig. S3C in the supplemental material). Stability was maintained up to 90 days on a family level (see Fig. S3D in the supplemental material). Microbiota composition of the IR was successfully transferred to and maintained in the TRs (see Fig. S3D in the supplemental material). Both metabolic activity (see Fig. S2C to E in the supplemental material) and composition were reproduced in the TRs.

10.1128/mSystems.01085-20.3 FIG S2 Concentrations (millimolar) of main SCFAs of PolyFermS effluent samples inoculated with immobilized and planktonic *L. plantarum*: (A) IR1 inoculated with donor 1 immobilized fecal microbiota and immobilized *L. plantarum*; (B) IR2 inoculated with donor 2 immobilized fecal microbiota and connected treatment reactors (TRs) (C) TR1 (period 1), (D) TR1 (period 2), and (E) TR2 spiked with planktonic *L. plantarum*. Download FIG S2, TIF file, 0.1 MB. Copyright © 2021 Isenring et al. 2021 Isenring et al. https://creativecommons.org/licenses/by/4.0/ This content is distributed under the terms of the Creative Commons Attribution 4.0 International license.

10.1128/mSystems.01085-20.4 FIG S3 Microbial composition in relative abundance obtained by 16S rRNA amplicon sequencing. Composition at phylum (A and C) and family (B and D) level of *in vitro* proximal colon microbiota of (A and B) IR1 inoculated with donor 1 immobilized fecal microbiota and immobilized *L. plantarum* and of (C and D) IR2 inoculated with donor 2 immobilized fecal microbiota and connected treatment reactors (TRs) TR1 (period 1), TR1 (period 2), and TR2 spiked with planktonic *L. plantarum*. Values at family level of <1% are summarized in the “Others” group. *x*-axis labeling indicates the day during fermentation relative to the start of the IR. Download FIG S3, TIF file, 0.2 MB. Copyright © 2021 Isenring et al. 2021 Isenring et al. https://creativecommons.org/licenses/by/4.0/ This content is distributed under the terms of the Creative Commons Attribution 4.0 International license.

### Prolonged cultivation of *L. plantarum* in *in vitro* human gut microbiota.

**FIG 2:**
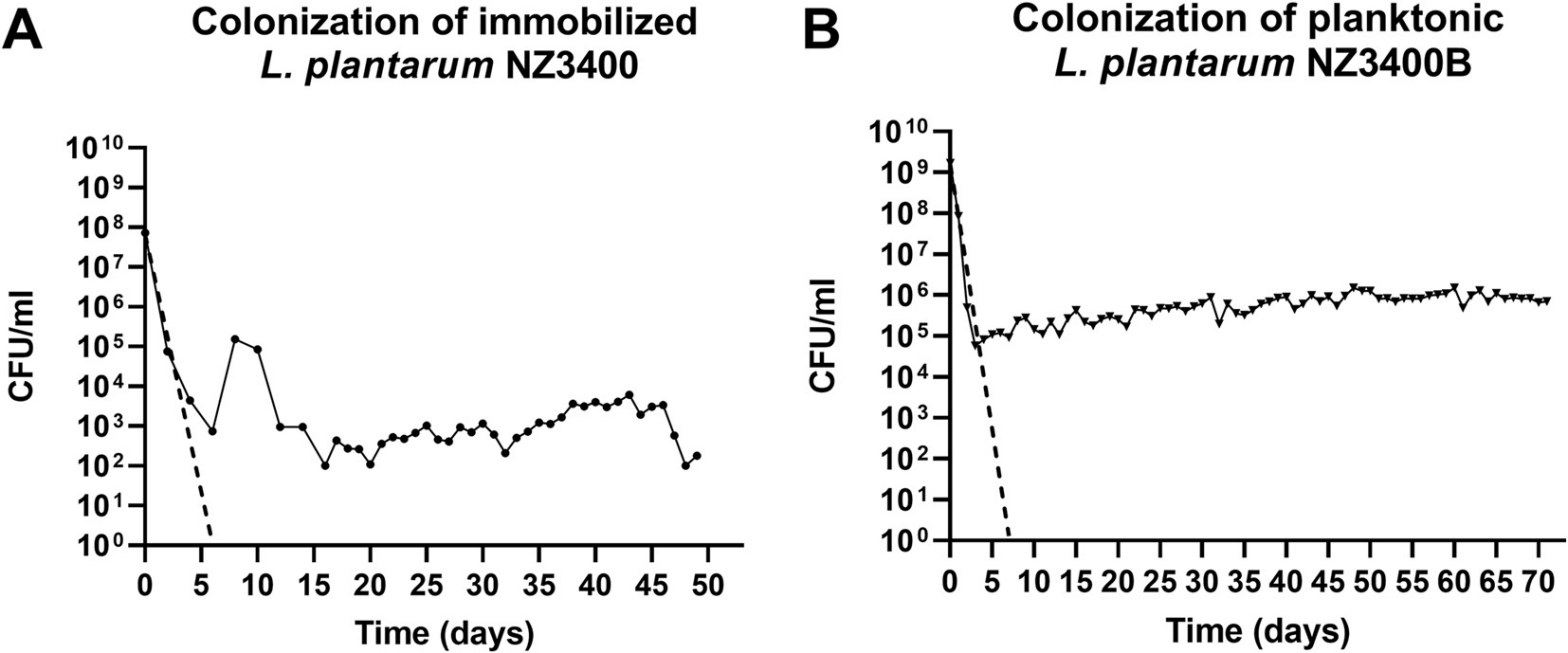
Colonization levels of *L. plantarum* during immobilized and planktonic adaptation in adult gut microbiota. (A) Immobilized *L. plantarum* NZ3400 (4 g of colonized beads containing 5 × 10^9^ CFU *L. plantarum*/g) was added to the gut microbiota (●) and (B) NZ3400B at 10^9^ CFU/ml effluent in planktonic state (▼). Dashed line indicates the theoretical washout of the system. Cell count is depicted on the *y* axis, and days of cultivation in the microbiota are shown on the *x* axis, where 0 corresponds to the day of *L. plantarum* supplementation.

To investigate the potential of the continuous *in vitro* gut fermentation model PolyFermS for evolutionary engineering, immobilized *L. plantarum* NZ3400 was added to the stabilized microbiota in IR1 at an initial concentration of 10^8^ CFU/ml effluent. NZ3400 decreased at the rate of the theoretical washout during the first 4 days (Fig. 2A), followed by colonization between 10^2^ CFU/ml and 10^4^ CFU/ml during the 50-day fermentation. *L. plantarum* was able to maintain a self-sustaining population, an observation which will be referred to as colonization.

The PolyFermS model was further evaluated for long-term adaptation of planktonic *L. plantarum* NZ3400B in TR2, fed by donor 2 gut microbiota. Cell counts decreased from 10^9^ to 10^5^ CFU/ml during the first 4 days after spiking (Fig. 2B), at the rate of the washout. Thereafter, colonization steadily increased to 10^6^ CFU/ml during 72 days. Repeated supplementation of *L. plantarum* with 10^6^ and 10^9^ CFU/ml in TR1 (period 1) and TR1 (period 2) resulted in stable colonization at different levels of 1 × 10^6^ and 3 × 10^4^ CFU/ml, respectively. Therefore, *L. plantarum* colonization at a donor-specific level was demonstrated for more than 50 days and 150 generations.

### Recovered *L. plantarum* strains are phenotypically adapted to the gut microbiota environment.

To test for *L. plantarum* adaptation during gut microbiota cultivation, recovered strains were grown in SCFA concentrations comparable to those in the gut fermenter. Average growth of strains from immobilized adaptation measured by optical density (OD) was impaired in De Man, Rogosa, and Sharpe (MRS) and MRS supplemented with 30 mM propionate or butyrate compared to the reference strain NZ3400B (Table 1). Strains from the early stage of long-term planktonic adaptation behaved similarly to the reference NZ3400B in all tested media. However, strains from late planktonic adaptation and biofilm grew better in MRS plus acetate (+0.11 and +0.07, respectively), less in standard MRS (−0.14 and −0.11, respectively), and similarly in MRS plus propionate or butyrate (Table 1) compared to NZ3400B. Moreover, strains from biofilm and late planktonic adaptation grew significantly better in the reactor-effluent-mimicking effluent-MacFarlane-sugar (EMS) medium than strains from early adaptation (Table 1).

**TABLE 1:** Growth of recovered *L. plantarum* strains in different media^*a*^

*L. plantarum* origin	MRS	MRS + acetate (50 mM)	MRS + propionate (30 mM)	MRS + butyrate (30 mM)	EMS
Immobilized adaptation	1.25 ± 0.11 c#	1.02 ± 0.15 cb	1.00 ± 0.24 b#	0.84 ± 0.24 c#	ND
Early planktonic adaptation	1.46 ± 0.05 b	1.06 ± 0.05 b	1.22 ± 0.03 a	1.22 ± 0.02 a#	0.69 ± 0.05 a#
Late planktonic adaptation	1.34 ± 0.05 a#	1.17 ± 0.06 a#	1.25 ± 0.07 a	1.21 ± 0.07 a	0.74 ± 0.04 b#
Biofilm of planktonic adaptation	1.37 ± 0.07 a#	1.13 ± 0.09 a#	1.22 ± 0.07 a	1.15 ± 0.06 b#	0.77 ± 0.05 c#
NZ3400B	1.48 ± 0.04	1.06 ± 0.04	1.23 ± 0.03	1.20 ± 0.03	0.63 ± 0.07

a The values reported are OD_600nm_ after 24 h. Values obtained for NZ3400B represent mean ± standard deviation from biological triplicates. All other values represent mean ± standard deviation for all strains recovered from one adaption period, whereas each recovered strain was measured in biological triplicates. Immobilized adaptation, *n* = 11 strains; early planktonic adaptation, *n* = 14 strains; late planktonic adaptation, *n* = 19 strains; biofilm of planktonic adaptation, *n* = 25 strains. Statistical significance is indicated as follows: a,b,c, significantly different from each other (*P* > 0.05 in a paired-sample *t* test, adjusted for unequal variance or normal distribution when needed); #, significant differences between NZ3400B and *L. plantarum* groups (one-sample *t* test, *P* ≤ 0.05). Abbreviations: ND, not determined; EMS, effluent-MacFarlane-sugar medium.

**FIG 3:**
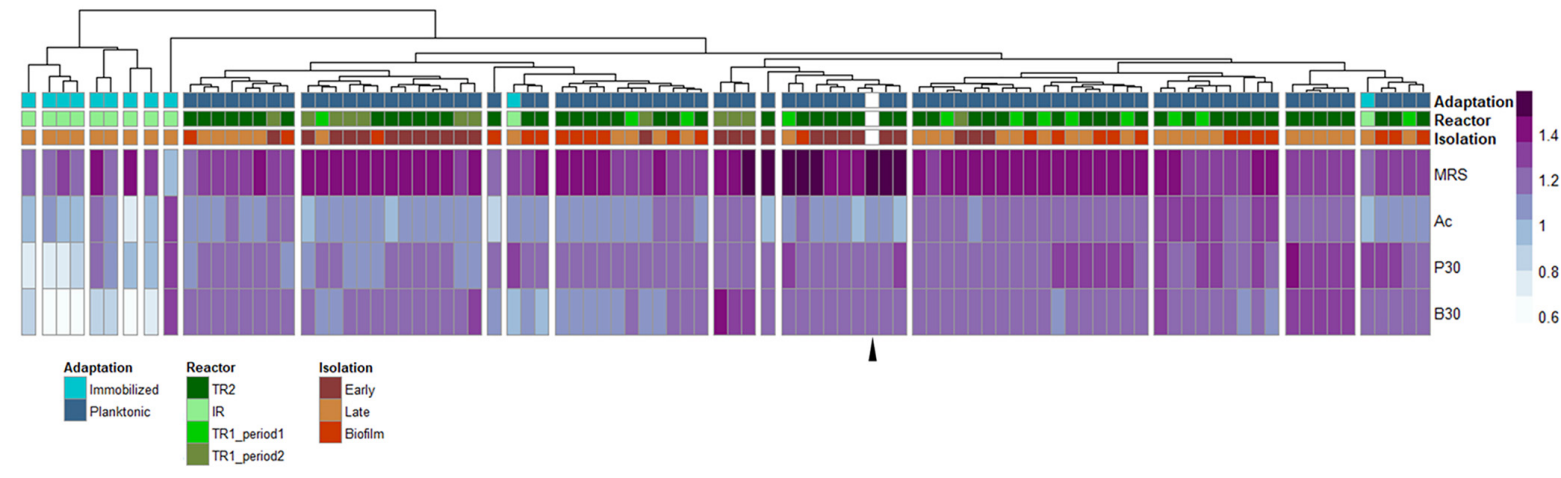
Heatmap visualization of growth behavior of potential mutant *L. plantarum* strains versus their reference strain NZ3400B in modified MRS medium (single combination of MRS with acetate [50 mM], butyrate [30 mM], or propionate [30 mM]). Symbols are as follows: (i) “Adaptation” indicates strain origin from immobilized or planktonic adaptation trials; (ii) “Reactor” describes the general experiment type for long-term planktonic adaptation (TR2) and the repetition of planktonic adaptation in TR1 (period 1) and TR1 (period 2); (iii) “Isolation” indicates biofilm/time point of isolation (early or late) of a strain during the adaptation cycles. Heatmap colors stand for measured ΔOD_600nm_ values after 24 h. The black triangle represents growth of *L. plantarum* NZ3400B. Ac, acetate; P, propionate; B, butyrate.

When clustered according to growth performance in MRS and MRS supplemented with acetate (50 mM), butyrate (30 mM), and propionate (30 mM), strains from immobilized and planktonic adaptation were clearly separated (Fig. 3). Further, strains recovered from immobilized adaptation exhibited a higher growth variability than strains isolated from the long-term planktonic adaptation (see Fig. S4 in the supplemental material). The reference NZ3400B clustered with strains isolated from early planktonic adaptation and clearly separate from strains isolated from effluent and biofilm at the end of adaptation (Fig. 3). Altogether, these results strongly hint toward adaptation of *L. plantarum* during prolonged cultivation in the gut microbiota. Furthermore, strains did not cluster according to the reactor they were isolated from, suggesting that the adaptation pattern is not dependent on the reactor but rather the time point of isolation.

10.1128/mSystems.01085-20.5 FIG S4 Principal-component analysis (PCA) of growth pattern of recovered *L. plantarum* strains. *L. plantarum* strains were recovered from the immobilized and planktonic adaptation cycle and grown in MRS and MRS supplemented with acetate (50 mM), propionate (30 mM), and butyrate (30 mM). Reference indicates the reference strain NZ3400B. Plot is based on the R function fviz_pca_ind. Download FIG S4, TIF file, 0.2 MB. Copyright © 2021 Isenring et al. 2021 Isenring et al. https://creativecommons.org/licenses/by/4.0/ This content is distributed under the terms of the Creative Commons Attribution 4.0 International license.

Phenotypes of these adaptations were stable for at least 190 generations (see Fig. S5 in the supplemental material). This strongly suggests that observed phenotypes are caused by mutations rather than physiological variations.

10.1128/mSystems.01085-20.6 FIG S5 Growth of *L. plantarum* in MRS during 4 weeks of daily serial cultivation. *L. plantarum* was serially cultivated in triplicates in MRS medium for 4 weeks. Final OD was measured in the first overnight culture (t0) and after 1, 2, 3, and 4 weeks (t1 to t4). ΔOD_600_ values were calculated by subtracting values obtained after 16 h of growth at 37°C by values obtained at the time point of inoculation. Values represent mean ± standard deviation for triplicates. The reference strain (RT) and selected recovered *L. plantarum* strains from immobilized and planktonic adaptation are depicted on the *x* axis. Download FIG S5, TIF file, 0.04 MB. Copyright © 2021 Isenring et al. 2021 Isenring et al. https://creativecommons.org/licenses/by/4.0/ This content is distributed under the terms of the Creative Commons Attribution 4.0 International license.

### Mutations in adapted *L. plantarum* strains hint toward adaptive evolution.

Stable altered phenotypes of recovered *L. plantarum* strains strongly suggest that these strains harbor mutations. Therefore, whole-genome sequencing of 45 strains randomly selected from adaptations experiments was performed. Comparison to the reference genome NZ3400B revealed 15 strains without any genotypic differences. Out of 18 single nucleotide polymorphisms (SNPs) confirmed by Sanger sequencing, two were detected in noncoding regions. The remaining 16 SNPs were found in genes involved in signaling, metabolism, transport, and cell surface (Table 2).

**TABLE 2:** Identified genotypic changes of recovered *L. plantarum* strains compared to NZ3400B^*f*^

SNP position^*a*^	SNP location within gene	AA change^*b*^	AA charge^*c*^	AA polarity^*d*^	Immobilized adaptation cycle											Planktonic adaptation cycle														Recovered *L. plantarum* IA_10					Locus tag^*e*^	Description
																TR1, period 2			TR2								TR1, period 1									
					IA01	IA02	IA03	IA04	IA05	IA06	IA07	IA08	IA09	IA10	IA11	PA1.2̱01	PA1.2̱02	PA1.2̱03	PA2̱01	PA2̱02	PA2̱03	PA2̱04	PA2̱05	PA2̱06	PA2̱07	PA2̱08	PA1.1̱01	PA1.1̱02	PA1.2̱03	IA̱10.1	IA̱10.2	A̱10.3	IA̱10.4	IA̱10.5		
					(i)	(i)		(ii)	(ii)	(ii)	(ii)	(ii)	(ii)	(ii)						(iii)	(iii)	(iv)	(iv)	(iv)												
3197325	C979T	Glu→Lys	− = +	P = P	x	x	x									x	x	x																	LP_RS14990	GHLK domain-containing protein
1441981	G645T	Met→Ile	N = N	A = A																								x							LP_RS06730	Glycerophosphodiester phosphodiesterase
2635801	A487C	Thr→Pro	N = N	P = A																									x						LP_RS12455	Lipase/esterase
2832369	C328T	Thr→Ile	N = N	P = A																									x						LP_RS13325	Class I SAM-dependent methyltransferase
3244391	C837A	Leu→Phe	N = N	A = A																		x	x	x							x				LP_RS15205	ROK family protein
945932	C2340T	None																x																	LP_RS04385	*rpoC*; DNA-directed RNA polymerase subunit beta
488529	G1192A	Asp→Asn	− = N	P = P																								x							LP_RS02260	Aminoacetyl-tRNA hydrolase
1536407	G881A	None																								x									LP_RS07205	*rsgA*; ribosome small subunit-dependent GTPase A
1737654	G173A	Ala→Val	N = N	A = A																							x								LP_RS08140	HAD_IC family P-type ATPase
3261513	C749T	None																	x	x	x														LP_RS15260	Extracellular solute-binding protein
1286590	A767G	Ile→Thr	N = N	A = P																						x									LP_RS05980	MFS transporter
300072	C995T	Thr→Met	N = N	P = A																							x								LP_RS01370	Acetoin ABC transporter permease
332157	G382A	Ala→Thr	N = N	A = P												x	x	x																	LP_RS01530	Hypothetical protein
2947006	G39T	Met→ Ile	N = N	A = A			x																												LP_RS13860	VanZ family protein
3025391	C569A	Gly→Val	N = N	A = A				x	x	x	x	x	x																	x	ϕ	x	x	x	LP_RS14255	WxL domain-containing protein
2307096	G984A	Met→Ile	N = N	A = A																											x				LP_RS10985	LPXTG cell wall anchor domain-containing protein
1116951		N.A.	N.A.	N.A.													x		x																gen. DNA	Intergenic; LP_RS05100 < LP_RS05095
67503		N.A.	N.A.	N.A.											x																				gen. DNA	Intergenic; LP_RS00275 < LP_RS00270

a Indicates SNP position in relation to the *L. plantarum* WCFS1 genome.

b Indicates amino acid change in the mutant compared to NZ3400B caused by the SNP. None, no change in amino acid; N.A., amino acid change not identifiable.

c Indicates change of the amino acid charge compared to NZ3400B caused by the SNP. N, neutral; +, positive charge; −, negative charge.

d Amino acid polarity. P, polar; A, apolar.

e Location of the SNP. Intergenic SNPs are labeled with genomic (gen.) DNA.

f “x” indicates an SNP; ϕ indicates loss of a mutation; Roman numerals in parentheses represent isogenic strains. AA, amino acid; SAM, *S*-adenosylmethionine; MFS, major facilitator superfamily.

The 11 mutated of 12 sequenced strains from the immobilized adaptation belonged to four different genotypes. Among these 11 strains, a mutation in the cell surface protein encoded by LP_RS14255 was found seven times. Further, a mutation in LP_RS14990, encoding a histidine kinase domain, was found three times (Table 2). Eight of 15 *L. plantarum* strains that were recovered at late stage of the long-term planktonic adaptation were mutated, resulting in five different genotypes (Table 2). Strikingly, an SNP in LP_RS14990 occurred independently during planktonic and immobilized adaptation. Moreover, the SNPs in LP_RS15205 and in the intergenic region between LP_RS05100 and LP_RS05095 were found in strains isolated from two different reactors.

Immobilized adaptation resulted in a bigger fraction of mutated strains, but they consisted predominantly of two isogenic lineages. Planktonic adaptation resulted in less frequent mutagenesis, yet higher mutant diversity. This suggests a difference in adaptation pressure, as already observed for the phenotypic screening. Recovery of some identical mutants from different adaptation experiments suggests that some of the observed mutations are involved in adaptation to the gut microbiota.

### Mutations in LP_RS14990 and LP_RS15205 are beneficial for *in vitro* gut microbiota colonization.

The mutation in the histidine kinase protein gene LP_RS14990 and the ROK protein gene LP_RS15205 occurred independently more than once in adaptation experiments. We therefore tested the fitness of each of the mutants *L. plantarum* PA2_06 (C837A in LP_RS15205), IA01 (C979T in LP_RS14990), or PA1.2_01 (C979T in LP_RS14990 and C837A in LP_RS15205) in competition experiments with the reference strain NZ3400B. Ten days of cultivation resulted in an increased abundance of all tested mutants in the gut microbiota compared to NZ3400B (Table 3).

**TABLE 3:** Increase in relative abundance (in %) of *L. plantarum* mutants during 10 days of competition against NZ3400B in *in vitro* human gut microbiota^*a*^

		*L. plantarum* mutant strains		
		IA01	PA2_06	PA1.2_01
Donor 2	Replicate 1	38 ± 5		
	Replicate 2	25 ± 1		
				
Donor 3	Replicate 1	66 ± 1	26 ± 3	11 ± 2
	Replicate 2	51 ± 1	31 ± 3	39 ± 2
				
Donor 4	Replicate 1		20 ± 2	
	Replicate 2		6 ± 2	

a Presented values show the increase of the ratio of *L. plantarum* mutant to the reference strain NZ3400B after 10 days of cultivation compared to the time point of inoculation. Values represent mean ± standard deviation for three DNA samples isolated at the same time point from the same reactor.

Pyrosequencing indicated that donor 2 gut microbiota had an *L. plantarum* background. Remarkably, the pyrogram of this background at the position LP_RS15205 C837A was identical to the Pyrogram of a sample containing NZ3400B and PA2_06. This shows that the nucleotide variation of both NZ3400B and PA2_06 also occurs naturally (see Fig. S6 in the supplemental material).

10.1128/mSystems.01085-20.7 FIG S6 Pyrogram obtained by pyrosequencing of the C837A SNP in LP_RS15205. (A) Pyrogram obtained from donor 2 gut microbiota prior to *L. plantarum* supplementation. (B) Pyrogram obtained from donor 3 gut microbiota supplemented with *L. plantarum* PA2_06 and NZ3400 in a 1:1 ratio. The *x* axis depicts the added nucleotide over time, and the *y* axis represents the light signal induced by incorporated nucleotides. Allele frequency was measured for G and T since pyrosequencing was done on the cDNA strand. Download FIG S6, TIF file, 0.09 MB. Copyright © 2021 Isenring et al. 2021 Isenring et al. https://creativecommons.org/licenses/by/4.0/ This content is distributed under the terms of the Creative Commons Attribution 4.0 International license.

### Mutation C979T in LP_RS14990 is stable under standard culturing conditions.

After observing increased fitness of mutants compared to the reference strain, it was investigated whether the mutations of *L. plantarum* IA01 in LP_RS14990 and *L. plantarum* PA2_06 in LP_RS15205 are stable during daily repeated batch cultures without the adaptation pressure of the gut microbiota. Stability of the LP_RS14990 mutation in the IA01 strain was observed during 12 batch cultures. However, the mutation C837A in strain PA2_06 was not stable since the reference strain nucleotide reoccurred at 3.5% ± 0.15% after 12 days (see Table S3 in the supplemental material). Investigation of NZ3400B in repeated MRS batch cultures revealed no occurrence of the SNPs of the mutants.

10.1128/mSystems.01085-20.9 TABLE S2 Primers used in this study. Download Table S2, DOCX file, 0.01 MB. Copyright © 2021 Isenring et al. 2021 Isenring et al. https://creativecommons.org/licenses/by/4.0/ This content is distributed under the terms of the Creative Commons Attribution 4.0 International license.

10.1128/mSystems.01085-20.10 TABLE S3 SNP stability in *L. plantarum* IA10 and PA2_06 during 12 days of continuous cultivation in MRS medium. Download Table S3, DOCX file, 0.01 MB. Copyright © 2021 Isenring et al. 2021 Isenring et al. https://creativecommons.org/licenses/by/4.0/ This content is distributed under the terms of the Creative Commons Attribution 4.0 International license.

### LP_RS14990 gene replacement in *L. plantarum* NZ3400B results in delay of gut microbiota colonization.

**FIG 4:**
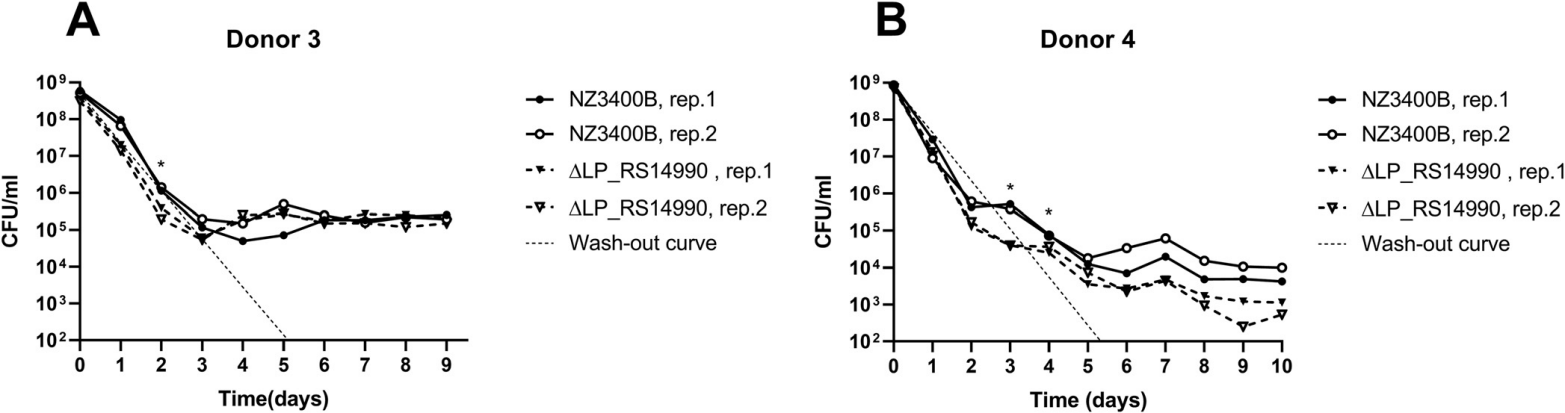
Colonization of ΔLP_RS14990 compared to the reference strain NZ3400B. *L. plantarum* strains were added to a level of 10^9^ CFU/ml reactor effluent. (A) *L. plantarum* ΔLP_RS14990 and NZ3400B were added into each of the two reactors (rep.1, rep.2) in donor 3 gut microbiota. (B) *L. plantarum* ΔLP_RS14990 and NZ3400B were added into each of the two reactors (rep.1, rep.2) in donor 4 gut microbiota. Dotted lines indicate the theoretical washout curve of the system. Cell count is depicted on the *y* axis, and days of cultivation in the microbiota are shown on the *x* axis, where 0 corresponds to the day of *L. plantarum* supplementation. *, significantly different colonization levels between NZ3400B and ΔLP_RS14990 (*P* ≤ 0.05, paired-sample *t* test).

To investigate the role of the LP_RS14990 gene in gut microbiota colonization, a ΔLP_RS14990 gene replacement strain was constructed and its colonization was compared to NZ3400B. NZ3400B started to colonize the gut microbiota of donor 3 on day 1 and donor 4 at day 3 since levels were above the washout curve (Fig. 4). Levels of ΔLP_RS14990 in both gut microbiota decreased more rapidly than the washout curve until day 3, suggesting cell death (Fig. 4). Strain ΔLP_RS14990 started to colonize the gut microbiota of both donors only after 4 days, later than the reference strain.

### In silico analysis of LP_RS15205 in *L. plantarum*.

Occurrence of the SNP C837A (L279F) in LP_RS15205 in *L. plantarum* strains from different reactors and in the background of the gut microbiota suggests an important function of this SNP in survival of *L. plantarum* in the gut microbiota. In silico analysis of LP_RS15205 revealed that C837A lies in a conserved domain with an ANLxGA motif that is found in *Firmicutes* and *Gammaproteobacteria* (data not shown). The function of this domain is unknown, but its conservation throughout two phyla strongly suggests that it might be important for protein function, and the mutation ANLxGA to ANFxGA might have an impact on protein functionality.

## DISCUSSION

Strain improvement is of major importance for food industries and probiotic cultures. Classical evolutionary adaptation approaches focus on specific strain characteristics in abiotic environments like improved performance of starter cultures or lactic acid bacteria (36, 37). However, for probiotics, improvement of gut colonization needs to consider abiotic and biotic factors. Here, we examined the suitability of the *in vitro* gut fermentation model PolyFermS to provide a gut microbiota environment for evolutionary engineering of *L. plantarum* NZ3400 toward a human adult gut microbiota. Immobilization of the donor gut microbiota and cultivation in the PolyFermS allowed reproduction of distinct gut microbiota representative for human adults (38, 39). The achieved long-term metabolic and compositional stability allowed the generation of adapted mutants, and the use of several donors increased the external validity of results on observed adaptations. The *L. plantarum* supplementation method and donor microbiota seem to influence phenotypic and genotypic adaptations.

Even though observed phenotypic differences of recovered *L. plantarum* were small, *L. plantarum* strains recovered from the immobilized adaptation showed very limited adaptation in contrast to recovered strains from planktonic adaptation trials, although abiotic conditions were highly similar. It thus is assumed that differences in adaptation patterns are caused by the supplementation method. Immobilization protects against stress and entraps cells physically in the system, leading to decreased adaptation pressure (40–42), which is in accordance with our phenotypic screening where strains from immobilized adaptation showed less adaptation. Polymer beads create mucosa-like attachment sites (43), and growth in beads results in a gradual release of sessile cells from the surface that then grow as planktonic cells in the bulk medium (44). The result is a mixed population consisting of cell lineages undergoing various numbers of growth cycles in the planktonic state. This might explain the more diverse phenotypic adaptation pattern among strains from the immobilized adaptation. It was further shown that immobilized cells are genetically more stable than planktonic cells (45–48), which was observed in our study where recovered strains from the immobilized adaptation mainly consisted of two isogenic lineages.

Two observations suggest that the observed genotypic changes in isolated strains were involved in adaptive evolution. The first is the known function of several of the genes affected by SNPs. Activated genes of *L. plantarum* in the murine digestive tract were involved in carbohydrate transport, metabolism and cell surface, sugar-related functions, molecule biosynthesis, and stress response (49, 50). Further, exposure of *L. plantarum* to the murine intestinal tract predominantly resulted in mutations in genes encoding cell wall-associated proteins (16). The SNPs identified in our study belong to the categories mentioned above. Remarkably, the latter study identified an SNP in a glycerophosphodiester phosphodiesterase-encoding gene, a gene also affected by an SNP in a recovered strain in our study. In summary, the function of mutated genes is in agreement with previously observed responses of *L. plantarum* to the *in vivo* intestinal environment. This shows similar selection pressures in the PolyFermS system as in *in vivo* settings.

The second observation indicating adaptive evolution in our experiment is that three mutations were found independently in multiple experiments. Since mutations occur continuously, −80°C stock cultures are rarely isogenic, and the mutants found in our study could have been already present as subculture in the NZ3400 stock (16). To counteract this bias, two different stock cultures, NZ3400 and NZ3400B were used for the immobilized and planktonic adaptation, respectively. Occurrence of identical mutants recovered from immobilized and planktonic adaptation with the SNP in LP_RS14990 seems therefore unlikely to originate from a stock subculture, as confirmed by Sanger sequencing (data not shown). Furthermore, the mutation was not detected by pyrosequencing of NZ3400B before inoculation (data not shown), minimizing a role for subcultures in our experiments. Moreover, the increased fitness of mutants shows the beneficial effect of detected mutations. This strongly supports that observed mutations are caused by adaptive evolution and demonstrates the suitability of the PolyFermS model to select relevant mutations related to the human gut microbiota. The exact nature of the evolutionary pressure in our system is not known and remains to be elucidated. It is likely the result of a combination of factors such as the competition for nutrients, metabolic cross-feeding, physiochemical factors, or presence of antimicrobial metabolites.

In conclusion, we demonstrated successful application of the continuous PolyFermS gut fermentation model to provide a long-term stable gut microbiota to generate adapted mutants to this environment. Immobilization of strains not only allows adaptive evolution of noncolonizers but also creates a culture consisting of sessile and planktonic cells mimicking the human gastrointestinal tract. The conditions of the model can be easily changed to other needs and selective pressures, including the source of microbiota and abiotic conditions. This novel technology enables identification of genes involved in gut microbiota colonization, persistence, and metabolism. The PolyFermS system could further be designed and applied for different fermentations, to trace and identify evolutionary and ecological processes between an exogenous single strain and a complex ecosystem.

## MATERIALS AND METHODS

### Bacterial strains and growth conditions.

Bacterial strains used during this study are listed in Table S1 in the supplemental material. *L. plantarum* NZ3400 (51) was used as reference strain. NZ3400 is a derivative of WCFS1, harboring a CM resistance cassette (P_32_-*cat*) in a neutral locus on the chromosome. *L. plantarum* was grown in De Man, Rogosa, and Sharpe (MRS; Labo-Life Sàrl, Pully, Switzerland) broth at 37°C, overnight. *L. plantarum* viable cells were enumerated by plating on MRS agar supplemented with CM (10 μg/ml), with aerobic incubation at 37°C, overnight.

10.1128/mSystems.01085-20.8 TABLE S1 Strains and plasmids used in this study. Download Table S1, DOCX file, 0.02 MB. Copyright © 2021 Isenring et al. 2021 Isenring et al. https://creativecommons.org/licenses/by/4.0/ This content is distributed under the terms of the Creative Commons Attribution 4.0 International license.

### Immobilization of adult fecal microbiota.

Fecal samples were obtained from four healthy adult individuals (27 to 31 years old) who did not take antibiotics and probiotics for at least 3 months and did not show detectable microbial growth on MRS+CM plates to avoid interference with *L. plantarum* NZ3400 recovery. Out of 15 tested fecal samples, four samples that did not show microbial growth on MRS+CM were chosen for fermentation (donors 1 to 4). The Ethics Committee of ETH Zürich exempted this study from review because the sample collection procedure has not been performed under conditions of intervention. Informed written consent was obtained from fecal donors. Fecal samples were immediately transferred to an anaerobic chamber within 2 h after defecation and suspended at 20% (wt/vol) in reduced peptone water (0.1%, pH 7; Thermo Fisher Diagnostics AG, Pratteln, Switzerland). Immobilization of 10 ml fecal slurry in polymer gel beads (gellan gum [2.5%, wt/vol], xanthan [0.25%, wt/vol], and sodium citrate [0.2%, wt/vol]) was performed as previously described (21, 44). The inoculum bioreactor (IR) (Sixfors; Infors, Bottmingen, Switzerland) was filled with 140 ml of vitamin-supplemented MacFarlane medium (Text S1), formulated to mimic the chyme entering the colon (20, 52), and inoculated with 60 ml of fecal gel beads. Beads were colonized in two fed-batch fermentations carried out at 37°C, pH 5.8, by controlled addition of NaOH (2.5 M), stirring at 180 rpm, and replacing 100 ml of spent medium with fresh medium after 16 h (21). Anaerobiosis was set by purging the bioreactor headspace with CO_2_ and monitored by redox potential probes.

10.1128/mSystems.01085-20.1 TEXT S1 Supplemental materials and methods. Download Text S1, DOCX file, 0.02 MB. Copyright © 2021 Isenring et al. 2021 Isenring et al. https://creativecommons.org/licenses/by/4.0/ This content is distributed under the terms of the Creative Commons Attribution 4.0 International license.

### Proximal colon fermentation in the PolyFermS model using immobilized human gut microbiota.

Continuous proximal colonic *in vitro* fermentations with immobilized human adult gut microbiota were performed in bioreactors as reported previously (26, 53). All fermentations were operated to mimic the adult proximal colon as described above for batch fermentation. Fresh medium was continuously added to the IR (25 ml/h), and fermented medium was removed to maintain a working volume of 200 ml. Because short-chain fatty acids (SCFAs) are the main fermentation end products of the gut microbiota, their stable production is a convenient, easily measurable marker for stability of continuous intestinal fermentation models (21, 54). The IR was run in continuous mode for at least 10 days to reach metabolic stability indicated by lower than 10% day-to-day variation (25, 54) in SCFA concentration, before connecting second-stage TRs. TRs were inoculated by the IR at 1.25 ml/h and fed at 23.75 ml/h. TRs were operated continuously for 4 days prior to *L. plantarum* supplementation in order to establish a gut microbiota activity akin to the IR. Addition of planktonic or immobilized *L. plantarum* was tested in IR and TRs with different donor microbiota (Fig. 1). In the case of two successive treatment periods in the same reactor, the reactor was disconnected after the first treatment period, sterilized, reconnected, and stabilized for 4 days before starting a second treatment.

Reactor effluent samples were taken daily to monitor the fermentation process, centrifuged for 10 min at 14,000 × *g*, 4°C, and stored at −20°C. Pellets were used for DNA extraction, and supernatants were used for metabolite analysis.

### Adaptive evolutionary engineering of *L. plantarum*. (i) Adaptive evolution using immobilized *L. plantarum* NZ3400.

Adaptive evolution of immobilized *L. plantarum* NZ3400 was tested in a continuously run single-stage IR (IR1) inoculated with donor 1 fecal beads (Fig. 1A). An *L. plantarum* overnight culture was harvested at 4°C, 4,000 × *g*, for 10 min and washed twice in phosphate-buffered saline (PBS), pH 6.2. Immobilization of *L. plantarum* was done as described for fecal samples under aerobic conditions. Beads were colonized during two pH-controlled batch cultures at 37°C for 16 h with stirring at 150 rpm. Colonized beads were washed in PBS, supplemented with cryoprotective buffer (55), and stored at −80°C. Before use, *L. plantarum* beads were reactivated during two batch cultures as described for colonization and washed twice in PBS. *L. plantarum* viable cell counts were determined by crushing 1 g of beads in PBS with a spatula and plating serial dilutions. Four grams of beads (5 × 10^9^ CFU *L. plantarum*/g) was added to the single-stage IR1 and cultivated for 53 days.

### (ii) Adaptive evolution using planktonic *L. plantarum* NZ3400B.

Due to microbial growth on MRS+CM plates of donor 1 fecal sample at this time, a new donor was chosen for the planktonic adaptation. To prevent carryover of possible mutants present in the initial NZ3400 stock culture, a new stock from a single NZ3400 colony isolate, designated NZ3400B was produced. NZ3400B was subjected to PacBio sequencing and used for all subsequent planktonic supplementation trials. NZ3400 and NZ3400B differed in eight SNPs but none thereof in SNP-affected genes of recovered *L. plantarum*. Adaptation of planktonic *L. plantarum* NZ3400B was tested in TRs continuously inoculated by the effluent from IR2 containing beads with immobilized fecal microbiota of donor 2 (Fig. 1B). Because the PolyFermS model was built with six TRs, two consecutive treatment periods were performed to test all treatments. *L. plantarum* strains were grown overnight, harvested, washed twice in PBS, resuspended in MacFarlane medium, and added to the TRs to a final level of 10^9^ CFU *L. plantarum*/ml effluent. Long-term planktonic adaptation was tested in TR2 operated for 72 days and repeated in TR1 (period 1) and TR1 (period 2) for 44 and 23 days, respectively (Fig. 1B).

Furthermore, the strain IA10, recovered from the immobilized adaptation, was added in planktonic state to TR3 (period 1) and TR3 (period 2) containing donor 2 gut microbiota to investigate effects of adaptations that occurred during the immobilized adaptation (Fig. 1B).

### Metabolite analysis of the continuous colon fermentation.

Reactors were sampled daily for analysis of the SCFAs acetate, butyrate, and propionate; branched-chain fatty acids isobutyrate, isovalerate, and valerate; and intermediate metabolites lactate and formate (56). Concentrations were determined by high‐performance liquid chromatography (HPLC) as described previously (23).

### Microbial profiling by 16S rRNA gene amplicon sequencing.

Genomic DNA of fecal and effluent samples was extracted using the FastDNA Spin kit for soil (MP Biomedicals, Illkirch, France) according to the manufacturer’s instructions. The V4 region of the 16S rRNA gene was amplified with the primers 806R (5′-GGACTACHVGGGTWTCTAAT-3′) and 515F (5′-GTGCCAGCMGCCGCGGTAA-3′). Amplicons were barcoded PCR based. Library preparation and sequencing (Illumina, CA, USA) using an Illumina MiSeq flow cell with a V2 reagent kit for 2 × 250-bp paired-end Nextera chemistry supplemented with 10% PhiX were performed in collaboration with the Genetic Diversity Center (GDC; ETH Zürich, Switzerland).

Raw data obtained from 16S rRNA sequencing were processed using Cutadapt (57) and DADA2 pipeline (58) to obtain amplicon sequence variants. Taxonomy was assigned using the SILVA database (v.132) (59) (full method described in Text S1).

### Recovery of *L. plantarum* from the gut microbiota.

*L. plantarum* colonization was determined by plating on MRS+CM agar. The combination of the MRS selectivity for lactobacilli and enterococci together with aerobe incubation and presence of antibiotics allowed growth repression of all other bacteria. Data were compared to the theoretical washout curve determined for absence of growth, from the following equation: *c_t_* = *c*_0_ × *e*^(^*^t^*^/RT)^ (53), where *c*_0_ and *c_t_* represent cell concentration at time point zero and *t*, respectively, and RT corresponds to the retention time. Colonies were randomly picked, incubated in MRS+CM overnight, mixed 1:1 with 60% (vol/vol) glycerol (Sigma-Aldrich Chemie GmbH, Buchs, Switzerland), and stored at −80°C.

Natural biofilm formed in TR2, used for long-term planktonic adaptation, and the repetition experiment in TR1 (period 1). To recover *L. plantarum* from biofilms, the vessels were emptied and washed twice with PBS. Remaining biofilm was removed and homogenized with glass beads (5 mm; VWR International AG, Dietikon, Switzerland) in dilution solution containing 0.85% (wt/vol) NaCl and 0.1% (wt/vol) peptone from casein (VWR International AG, Dietikon, Switzerland). Dilutions were plated with subsequent strain recovery and storage performed as described above.

### Phenotypic characterization of recovered *L. plantarum* strains.

Growth behavior of recovered *L. plantarum* strains was analyzed in MRS supplemented with each of the main SCFAs of the human gut microbiota, acetate (50, 75, and 100 mM), butyrate (15, 30, and 45 mM), and propionate (15, 30, and 45 mM) in similar concentrations as measured during colonic fermentations (see Fig. S2 in the supplemental material). The abiotic gut fermentation environment was simulated in effluent-MacFarlane-sugar (EMS) medium consisting of filter-sterilized PolyFermS effluent, MacFarlane medium in a 9:1 ratio, and 0.75% (wt/vol) glucose (see Fig. S1 in the supplemental material). Glucose was added since *L. plantarum* was unable to grow in MacFarlane medium. For comparison of the effect on adaption in immobilized and long-term planktonic adaptation trials performed in TR2 (Fig. 1B), recovered strains were divided into four groups based on their origin of isolation: (i) 11 strains from the effluent at the late stage of immobilized adaptation after 53 days, (ii) 14 strains from the effluent during day 7 and day 23 (early planktonic adaptation), (iii) 19 strains from the effluent during day 60 and day 72 (late planktonic adaptation), and (iv) 25 strains from the biofilm of planktonic adaptation after 72 days. Strains were isolated at an early stage after seven (stable *L. plantarum* colonization) and 23 days and a late stage of 60 and 72 days to increase the chance to observe adaptation. Biofilm was sampled on the last day of fermentation because the reactor had to be emptied for biofilm sampling. Growth analysis was done in 96-well tissue culture test plates (Bioswisstec AG, Schaffhausen, Switzerland). Wells were filled with 200 μl of medium and inoculated with the potentially adapted *L. plantarum* at 37°C. Growth was monitored by optical density (OD) measurement at 600 nm in a plate reader after 24 h (PowerWaveTMXS; Bio-Tek Instrument Inc., Winooski, VT, USA) in biological triplicates.

10.1128/mSystems.01085-20.2 FIG S1 Comparison of metabolite concentrations in EMS medium and the reactor effluent. Bar plot representing metabolite concentrations (millimolar) of the EMS medium consisting of reactor effluent, MacFarlane medium, and glucose and the reactor effluent without *L. plantarum* supplementation. Samples were analyzed in biological triplicates. Download FIG S1, TIF file, 0.03 MB. Copyright © 2021 Isenring et al. 2021 Isenring et al. https://creativecommons.org/licenses/by/4.0/ This content is distributed under the terms of the Creative Commons Attribution 4.0 International license.

Phenotype stability was assessed by repeated daily culturing of strains in MRS for 28 days, approximately 190 generations, as presented above. Stability was measured after 1, 7, 14, 21, and 28 days, which corresponds to the time of transcriptome homogenization among *L. plantarum* strains isolated from different habitats (60).

### Complete genome sequencing and data analysis.

*L. plantarum* genomic DNA was isolated via lysozyme-based cell lysis (61) followed by purification using the Wizard Genomic DNA purification kit according to the manufacturer's instructions (Promega, Dübendorf, Switzerland). The genome of the reference strain NZ3400B was sequenced at the Functional Genomics Center Zurich (Zürich, Switzerland) on PacBio RS II (Pacific Biosciences, Menlo Park, CA, USA) using one SMRT cell. Reads were assembled using Hierarchical Genome Assembly Process (HGAP) assembly as described previously (62). All other *L. plantarum* strains were sequenced using Illumina MiniSeq (Illumina, CA, USA) with 250-bp paired-end reads at the Institute for Food Safety and Hygiene, University of Zurich (62). Reads were merged and mapped to the reference genome *L. plantarum* NZ3400B using CLC Genomic Workbench 11.0 (Qiagen CLC bio, Aarhus, Denmark) using default parameters. Single nucleotide polymorphisms (SNPs) were extracted using Parsnp (63). SNPs were confirmed by Sanger Sequencing (Eurofins GATC, Biotech GmbH, Constance, Germany). SNP-related changes in amino acid sequence were determined in CLC Genomic Workbench.

### Competition experiments in the human gut microbiota.

Competition experiments between NZ3400B and the mutant strains *L. plantarum* IA01, PA1.2_01, and PA2_06 were performed to determine the effect of the mutations on gut microbiota colonization (Fig. 1B). NZ3400B was paired with each of the three mutant strains in a 1:1 ratio to reach 10^9^ CFU/ml reactor effluent in the gut microbiota and cultivated for 10 days. Ten days was sufficient to obtain stable *L. plantarum* colonization for at least 4 days but also as briefly as possible to minimize the chance of proliferation of new mutants. Relative abundance of the strains in the complex community was determined by measuring allele frequency of the genes LP_RS14990 and LP_RS15205, respectively, via Pyrosequencing (full method described in Text S1).

### Stability of mutations under standard cultivation conditions.

Stability of the mutations in *L. plantarum* IA01 and PA2_06 was investigated during repeated daily cultures in MRS for 12 days, approximately 81 generations, performed in triplicates. Cultivation of NZ3400B served as control. Allele frequency was determined via pyrosequencing as described above.

### Plasmid construction and LP_RS14990 gene replacement of *L. plantarum* NZ3400B.

Six mutants carrying the mutation in LP_RS14990 were recovered from independent adaptation experiments. To investigate the involvement of this gene in gut microbiota colonization, an *L. plantarum* ΔLP_RS14990 knockout was constructed by double-crossover gene replacement in *L. plantarum* NZ3400B (full method in Text S1). The knockout strain was subsequently added to the gut microbiota, and colonization levels were compared to the reference strain NZ3400B (Fig. 1B).

### In silico analysis of LP_RS15205 in *L. plantarum*.

LP_RS15205 was affected by an identical mutation in four strains recovered from two independent adaptation experiments. Since this mutation was also found in the background gut microbiota, in silico analysis of this locus was performed. Complete genome sequences of *Firmicutes* (*n* = 557), *Bacteroidetes* (*n* = 218), *Actinomycetes* (*n* = 457), and *Gammaproteobacteria* (*n* = 638) from the NCBI genome database were downloaded in May 2020. The amino acid sequence of LP_RS15205 was subjected to a BLAST search against these genomes using standard settings, and significant hits were aligned using MUSCLE (64).

### Data analysis.

Statistics for growth experiments were calculated in R (version 3.6.2) using a one-sample *t* test for comparison to *L. plantarum* NZ3400B and a paired-sample *t* test for comparison within recovered *L. plantarum* groups. Values represent mean values ± standard deviations. The heatmap was generated using the R pheatmap package and Euclidean distance measure. Allele frequency determination by pyrosequencing was calculated based on three extracted DNA samples of the same time point. Graphs were created using GraphPad Prism version 8 (GraphPad Software Inc., San Diego, CA, USA).
