# Supplementary figures and images for: In Vitro Gut Modeling as a Tool for Adaptive Evolutionary Engineering of Lactiplantibacillus plantarum
Source: mSystems. 2021 Apr 13;6(2):e01085-20. doi: 10.1128/mSystems.01085-20 (PMC8546992; doi:10.1128/mSystems.01085-20)

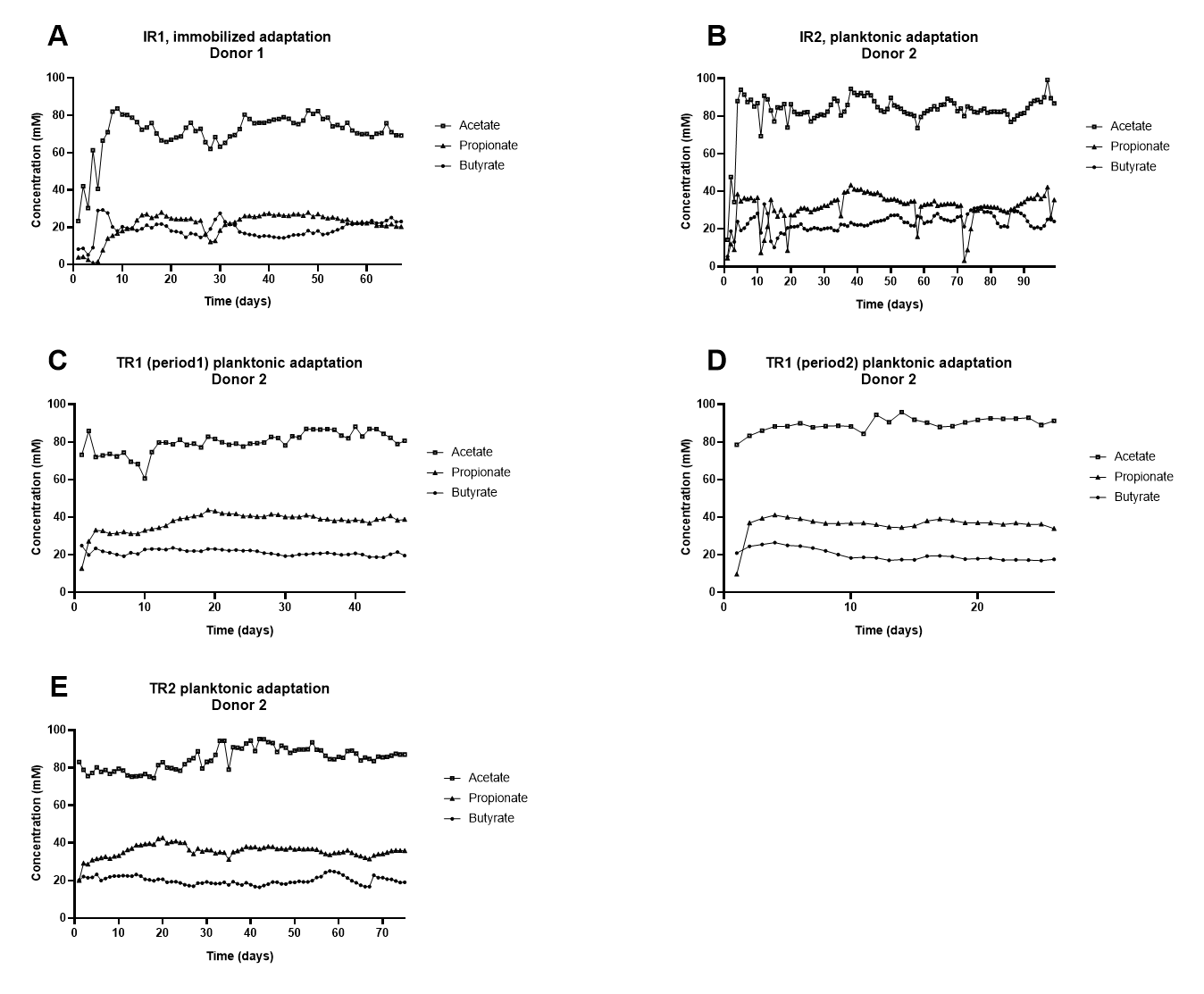

Supplement: FIG S2 [file msystems.01085-20-sf002.tif]

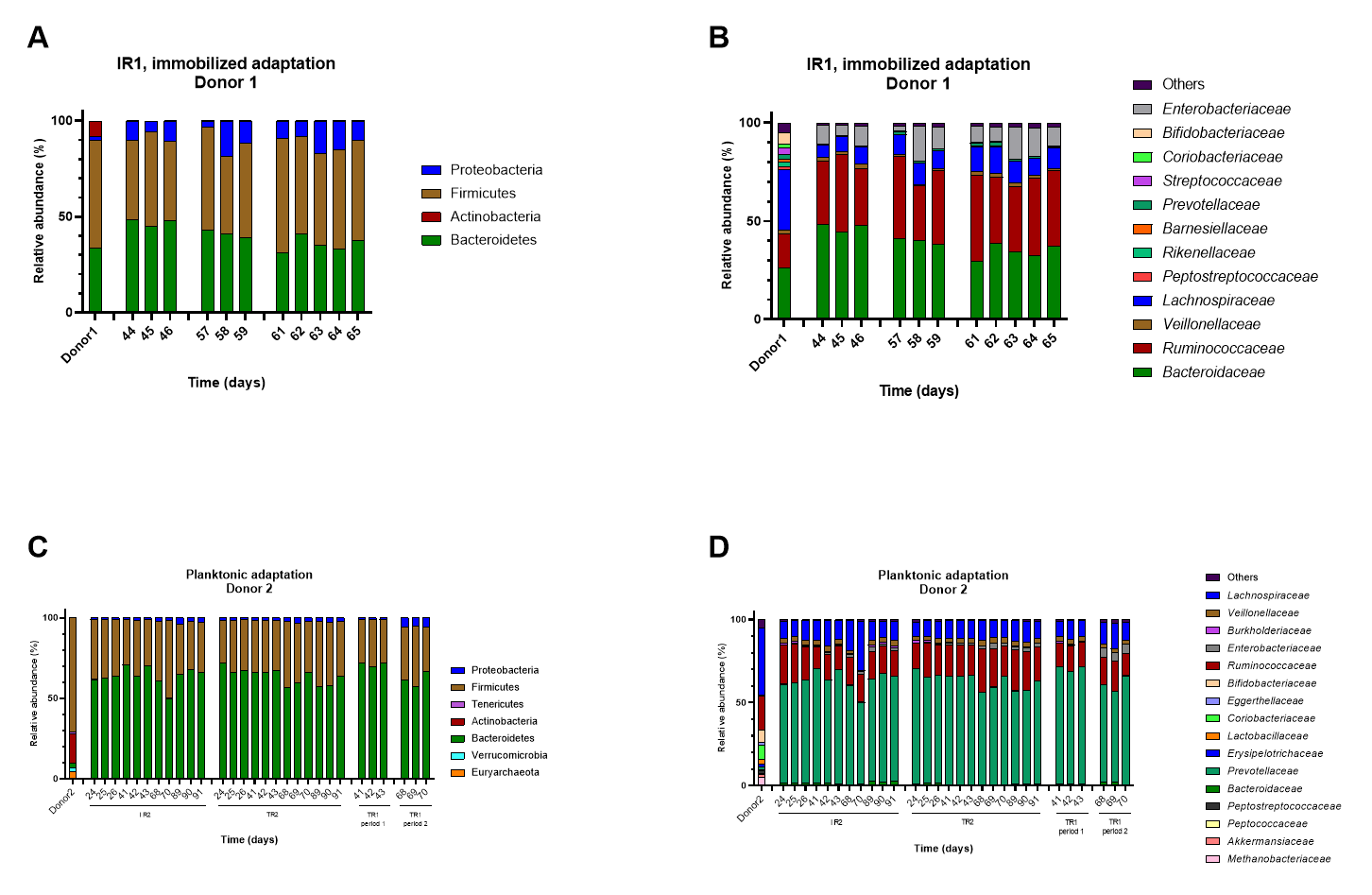

Supplement: FIG S3 [file msystems.01085-20-sf003.tif]

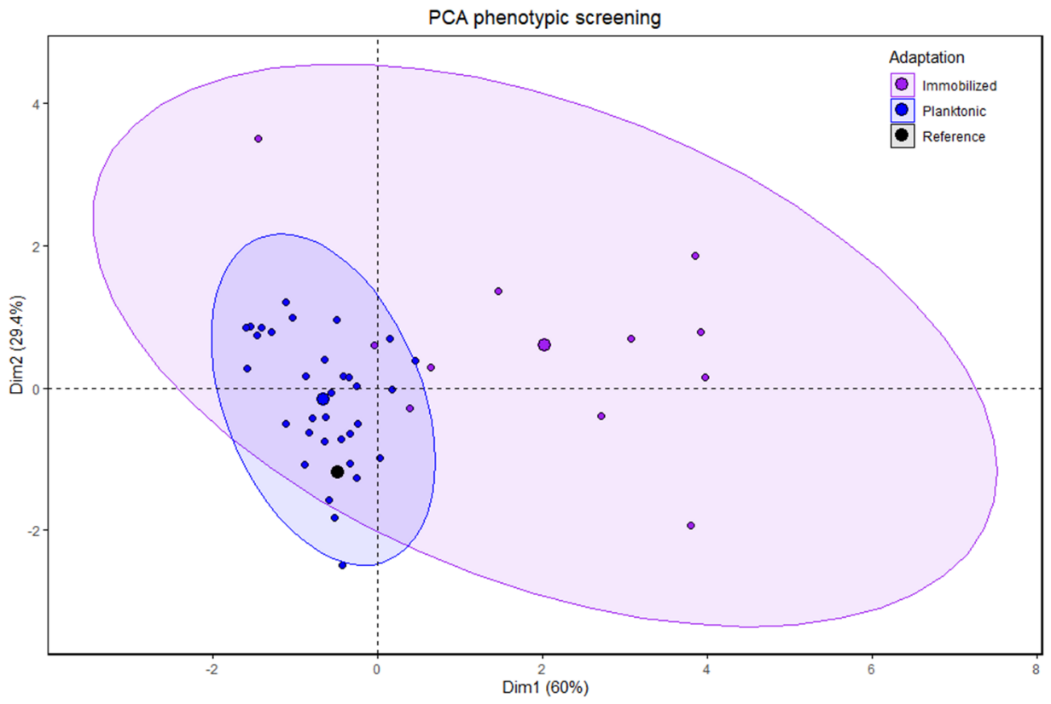

Supplement: FIG S4 [file msystems.01085-20-sf004.tif]

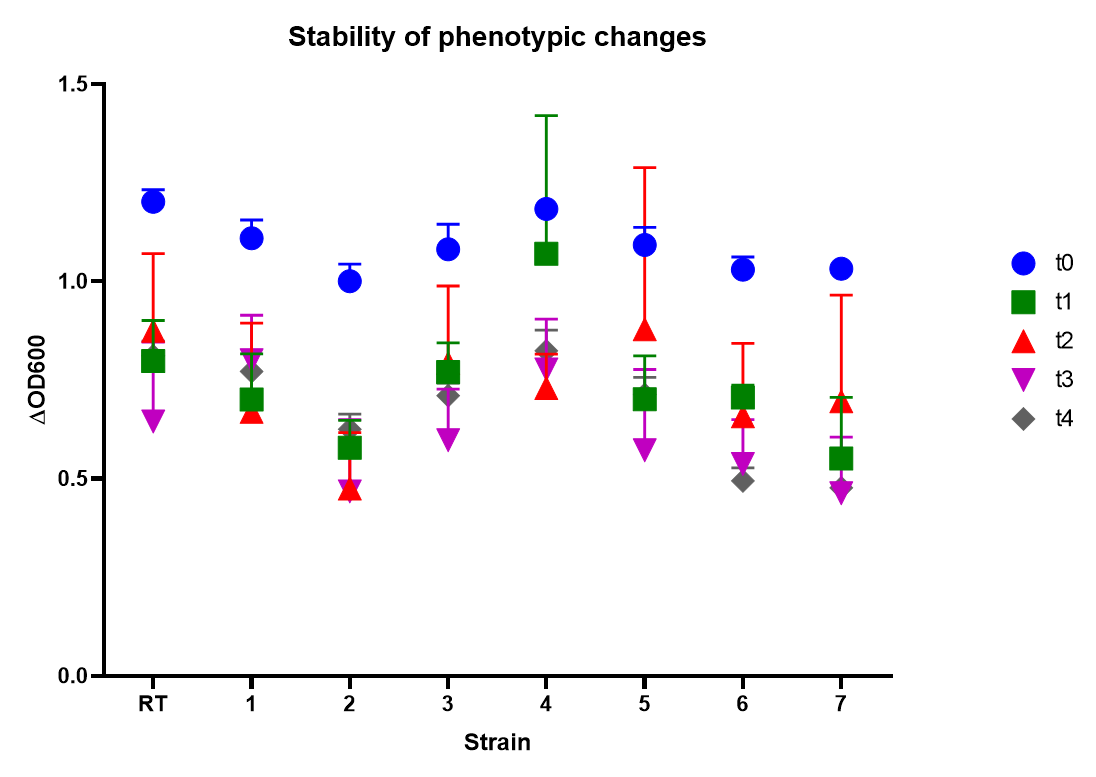

Supplement: FIG S5 [file msystems.01085-20-sf005.tif]

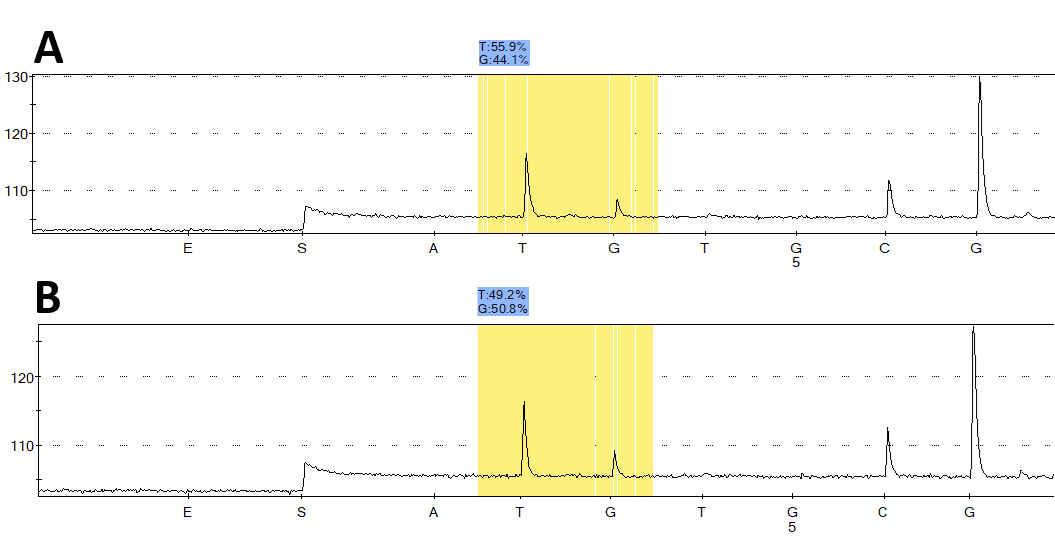

Supplement: FIG S6 [file msystems.01085-20-sf006.tif]

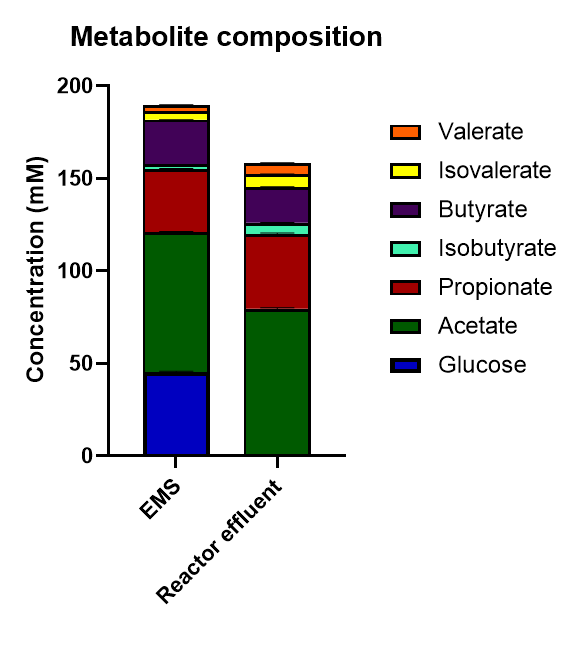

Supplement: FIG S1 [file msystems.01085-20-sf001.tif]
