# Supplementary material for: In Vitro Gut Modeling as a Tool for Adaptive Evolutionary Engineering of Lactiplantibacillus plantarum
Source: mSystems. 2021 Apr 13;6(2):e01085-20. doi: 10.1128/mSystems.01085-20 (PMC8546992; doi:10.1128/mSystems.01085-20)
Supplement: TABLE S2 [file msystems.01085-20-st002.docx]

**Supplementary Table S2**: Primers used in this study.

| **Name** | **Sequence (5'-3')** | **Description** |
| --- | --- | --- |
| lamC-5' | TGAGATACTCGCTTCACCGTC | Targeting LP_RS14990 |
| lamC-3' | ATGAGTTGCGACGTTTCAGAC | Targeting LP_RS14990 |
| lamC-D-5' | CGTCTACACCATGTTCGGTTA | Targeting flanking region of LP_RS14990 |
| lamC-U-3' | GAAGGCAATTCGGCGTAAC | Targeting flanking region of LP_RS14990 |
| ery-5' | GAATCGAGACTTGAGTGTG | Targeting *ery* gene |
| ery-3' | CACACTCAAGTCTCGATTC | Targeting *ery* gene |
| lamC-Pyro-D-5' | GGGTGGTAACTGTTCAAAAACATT | Pyrosequencing, downstream of the SNP C979T in LP_RS14990 |
| lamC-Pyro-U-3' | AGCTATGCACAATCGCAAGAGA | Pyrosequencing, upstream of the SNP C979T in LP_RS14990 |
| lamC-seq | TGTCCTACCGATGCTT | Pyrosequencing, sequencing primer LP_RS14990 |
| 3630-Pyro-D-5' | TGGGATTGTTCAAAATCAACTG | Pyrosequencing, downstream of the SNP C979T in LP_RS15205 |
| 3630-Pyro-U-3' | AGGTCAAGATTGCCACGTTAA | Pyrosequencing, upstream of the SNP C979T in LP_RS15205 |
| 3630-seq | CCGATGAAGCGAACT | Pyrosequencing, sequencing primer LP_RS15205 |
