# Supplementary material for: In Vitro Gut Modeling as a Tool for Adaptive Evolutionary Engineering of Lactiplantibacillus plantarum
Source: mSystems. 2021 Apr 13;6(2):e01085-20. doi: 10.1128/mSystems.01085-20 (PMC8546992; doi:10.1128/mSystems.01085-20)
Supplement: TABLE S3 [file msystems.01085-20-st003.docx]

**Supplementary Table S3**: SNP stability in *L. plantarum* IA10 and PA2_06 during 12 days of continuous cultivation in MRS medium.

|  | Allele frequency (%) at day 0 | | | Allele frequency (%) at day 12 | | |
| --- | --- | --- | --- | --- | --- | --- |
|  | Replicate 1 | Replicate 2 | Replicate 3 | Replicate 1 | Replicate 2 | Replicate 3 |
| LP_RS14990 gene ^a)^ |  |  |  |  |  |  |
| C | 0 | 0 | 0 | 0 | 0 | 0 |
| T | 100 | 100 | 100 | 100 | 100 | 100 |
|  |  |  |  |  |  |  |
| LP_RS15205 gene ^b)^ |  |  |  |  |  |  |
| G | 0 | 0 | 0 | 3.6 | 3.3 | 3.5 |
| T | 100 | 100 | 100 | 96.4 | 96.7 | 96.5 |
|  |  |  |  |  |  |  |

a) Allele frequency of the LP_RS14990 mutation C979T in *L. plantarum* IA01 culture.

b) Allele frequency of the LP_RS15205 mutation C837A in *L. plantarum* PA2_06 culture. Allele frequency was measured for G and T since Pyrosequencing was done on the complementary DNA strand.
