# Supplementary material for: In Vitro Gut Modeling as a Tool for Adaptive Evolutionary Engineering of Lactiplantibacillus plantarum
Source: mSystems. 2021 Apr 13;6(2):e01085-20. doi: 10.1128/mSystems.01085-20 (PMC8546992; doi:10.1128/mSystems.01085-20)
Supplement: TABLE S1 [file msystems.01085-20-st001.docx]

**Supplementary Table S1**: Strains and plasmids used in this study.

|  | **Relevant features** | **Source of reference** |
| --- | --- | --- |
| ***L. plantarum*** |  |  |
| NZ3400 | Reference strain, WCFS1 derivative containing a lox66-P32-cat-lox71 insertion in the neutral H-locus | Remus *et al.*, 2012 |
| IA_10.2 | Derivative strain of IA10 with C837A in LP_RS15205 and G984A in LP_RS10985, day 23, Cm^R^ | This study |
| IA_10.3 | Derivative strain of IA10 with C569A in LP_RS14255, day 23, Cm^R^ | This study |
| PA1.1_06 | Strain from biofilm of planktonic adaptation, TR1 (period1), day 44, Cm^R^ | This study |
| PA1.1_07 | Strain from biofilm of planktonic adaptation, TR1 (period1), day 44, Cm^R^ | This study |
| PA1.2_01 | Strain with C979T in LP_RS14990 and G382A in LP_RS01530 from biofilm of planktonic adaptation, TR1 (period2), day 23, Cm^R^ | This study |
| PA1.2_02 | Strain with C979T in LP_RS14990*,* G382A in LP_RS01530 and intergenic SNP LP_RS05100 < LP_RS05095 from biofilm of  planktonic adaptation, TR1 (period2), day 23, Cm^R^ | This study |
| PA1.2_03 | Strain with C979T in LP_RS14990*,* G382A in LP_RS01530 and C2340T in LP_RS04385 from biofilm of planktonic adaptation,  TR1 (period2), day 23, Cm^R^ | This study |
| PA2_03 | Strain with C749T in LP_RS15260 from biofilm of planktonic adaptation, TR2, day 72, Cm^R^ | This study |
| PA2_07 | Strain with A767G in LP_RS05980 from biofilm of planktonic adaptation, TR2, day 72, Cm^R^ | This study |
| PA2_08 | Strain with C995T in LP_RS01370 and G881A in LP_RS07205 from biofilm of planktonic adaptation, TR2, day 72, Cm^R^ | This study |
| PA2_13 | Strain from biofilm of planktonic adaptation, TR2, day 72, Cm^R^ | This study |
| PA2_14 | Strain from biofilm of planktonic adaptation, TR2, day 72, Cm^R^ | This study |
| PA2_15 | Strain from biofilm of planktonic adaptation, TR2, day 72, Cm^R^ | This study |
| IA_10.1 | Derivative strain of IA10 with C569A in LP_RS14255, day 23, Cm^R^ | This study |
| IA_10.4 | Derivative strain of IA10 with C569A in LP_RS14255, day 23, Cm^R^ | This study |
| IA_10.5 | Derivative strain of IA10 with C569A in LP_RS14255, day 23, Cm^R^ | This study |
| PA1.1_01 | Strain with G173A in LP_RS08140 from planktonic adaptation, TR1 (period1), day 44, Cm^R^ | This study |
| PA1.1_02 | Strain with G645T in LP_RS06730 and G1192A in LP_RS02260 from planktonic adaptation, TR1 (period1), day 44, Cm^R^ | This study |
| PA1.1_03 | Strain with A487C in LP_RS12455 and C328T in LP_RS13325 from planktonic adaptation, TR1 (period1), day 44, Cm^R^ | This study |
| PA1.1_04 | Strain from planktonic adaptation, TR1 (period1), day 41, Cm^R^ | This study |
| PA1.1_05 | Strain from planktonic adaptation, TR1 (period1), day 41, Cm^R^ | This study |
| PA2_01 | Strain with intergenic SNP LP_RS05100 < LP_RS05095 and C749T in LP_RS15260 from planktonic adaptation, TR2, day 64, Cm^R^ | This study |
| PA2_02 | Strain with C749T in LP_RS15260 from planktonic adaptation, TR2, day 72, Cm^R^ | This study |
| PA2_04 | Strain with C837A in LP_RS15205 from planktonic adaptation, TR2, day 72, Cm^R^ | This study |
| PA2_05 | Strain with C837A in LP_RS15205 from planktonic adaptation, TR2, day 72, Cm^R^ | This study |
| PA2_06 | Strain with C837A in LP_RS15205 from planktonic adaptation, TR2, day 72, Cm^R^ | This study |
| PA2_09 | Strain from planktonic adaptation, TR2, day 64, Cm^R^ | This study |
| PA2_10 | Strain from planktonic adaptation, TR2, day 72, Cm^R^ | This study |
| PA2_11 | Strain from planktonic adaptation, TR2, day 72, Cm^R^ | This study |
| PA2_12 | Strain from planktonic adaptation, TR2, day 72, Cm^R^ | This study |

Supplementary Table S1, continued

|  | **Relevant features** | **Source of reference** |
| --- | --- | --- |
| ***L. plantarum*** |  |  |
| IA01 | Strain with C979T in LP_RS14990 from immobilized adaptation, day 53, Cm^R^ | This study |
| IA02 | Strain with C979T in LP_RS14990 from immobilized adaptation, day 53, Cm^R^ | This study |
| IA03 | Strain with C979T in LP_RS14990 and G39T in LP_RS13860 from immobilized adaptation, day 53, Cm^R^ | This study |
| IA04 | Strain with C569A in LP_RS14255 from immobilized adaptation, day 53, Cm^R^ | This study |
| IA05 | Strain with C569A in LP_RS14255 from immobilized adaptation, day 53, Cm^R^ | This study |
| IA06 | Strain with C569A in LP_RS14255 from immobilized adaptation, day 53, Cm^R^ | This study |
| IA07 | Strain with C569A inLP_RS14255 from immobilized adaptation, day 53, Cm^R^ | This study |
| IA08 | Strain with C569A in LP_RS14255 from immobilized adaptation, day 53, Cm^R^ | This study |
| IA09 | Strain with C569A in LP_RS14255 from immobilized adaptation, day 53, Cm^R^ | This study |
| IA10 | Strain with C569A in LP_RS14255 from immobilized adaptation, day 53, Cm^R^ | This study |
| IA11 | Strain with intergenic SNP LP_RS00275 < LP_RS00270 from immobilized adaptation, day 53, CmR | This study |
| IA12 | Strain from immobilized adaptation, day 53, Cm^R^ | This study |
| Strain1-7 | Strains from planktonic adaptation, used to assess phenotypic stability |  |
| ***E. coli*** |  |  |
| MC1000 |  | Casadaban *et al*., 1980 |
| ***Plasmid*** |  |  |
| pUC18_lp_  lamC | Em^R^, gene replacement vector | This study |
|  |  |  |

Cm^R^: chloramphenicol-resistance, Em^R^: erythromycin-resistance
