# Supplementary material for: In Vitro Gut Modeling as a Tool for Adaptive Evolutionary Engineering of Lactiplantibacillus plantarum
Source: mSystems. 2021 Apr 13;6(2):e01085-20. doi: 10.1128/mSystems.01085-20 (PMC8546992; doi:10.1128/mSystems.01085-20)
Supplement: TEXT S1 [file msystems.01085-20-s0001.docx]

**Supplementary Data 1 – Methods selection**

## **MacFarlane medium composition and supplemented vitamins**

| **Component** | **g/l** | **Vitamin** | **µg/l** |
| --- | --- | --- | --- |
| Pectin (citrus)  Yeast extract  Xylan (oat spelts)  Arabinogalactan (larch wood)  Guar gum  Inulin (Raftiline® HP)  Soluble starch (potato)  Mucin  Casein acid hydrolysate  Peptone water  Bacto^TM^ Tryptone  L-Cysteine HCl  Bile Salts  KH_2_PO_4_  NaHCO_3_  NaCl  KCl  MgSO_4_ anh. (M: 120.37)  CaCl_2_*2H_2_O (M: 147.02)  MnCL_2_*4H_2_O (M: 197.91)  FeSO_4_*7H_2_O (M: 278.02)  Hemin solution  Tween 80  Vitamin solution | 2  4.5  2  2  1  1  5  4  3  5  5  0.8  0.4  0.5  1.5  4.5  4.5  0.61  0.1  0.2  0.005  1 ml  1 ml  1 ml | Pyridoxine-HCl (Vit. B_6_)  4-Aminobenzoic acid (PABA)  Nicotinic acid (Vit. B_3_)  Biotin (Vit. H), stock: 4mg/ml  Folic acid (Vit. B_9_), stock: 4mg/ml  Cyanocobalamine (Vit. B_12_)  Thiamine (Vit. B_1_HCl)  Riboflavine (Vit. B_2_)  Phylloquinone (Vit. K_1_), stock: 0.15 mg/ml  Menadione (Vit. K_3_), stock: 2 mg/ml  Pantothenate (Vit. B_5_) | 100  50  50  20  20  5  50  50  0.075  10  100 |
| Adjusted pH to 5.8  Filter-sterilized vitamin solution added after autoclaving | | | |

## **Microbial profiling by 16S rRNA gene amplicon sequencing**

Removal of Illumina adaptors and gene-specific primers was done using Cutadapt (1). Sequences were processed using the DADA2 pipeline (2) which allows inference of exact amplicon sequence variants (ASVs). Forward and reverse reads were truncated after 170 and 160 nucleotides, respectively. After truncation, reads with expected error rates higher than three and four for forward and reverse reads, respectively, were removed. After filtering, error rate learning, ASV inference, and denoising, reads were merged with a minimum overlap of 40 bp. Chimeric sequences were identified and removed. Taxonomy was assigned using the DADA2 formatted SILVA database (v.132) (3).

## **Competition experiments in the human gut microbiota**

Primers (Supplementary Table 2) selectively targeting *L. plantarum* in the gut microbiota up- and downstream of investigated SNPs and sequencing primers were designed as described previously (4) using PyroMark^TM^ID 1.0 software (Biotage AB and Biosystems, Uppsala, Sweden). To determine the background of the method, DNA isolated from gut microbiota before *L. plantarum* addition was screened. If an allele was absent in the gut microbiota, whole microbiota DNA was used. In case of background, exogenously added *L. plantarum* was enriched by adding 100 μl reactor effluent to 10 ml MRS + CM and grown at 37°C overnight. Cells were harvested and DNA was isolated using the FastDNA® SPIN Kit for Soil.

Biotin amplification by PCR was performed using the 2X PCR Master Mix (Life Technologies Europe BV, Zug, Switzerland) and 40 ng DNA in a T3000 Thermocycler (Biometra, Göttingen, Germany) as described previously (4). Immobilization on streptavidin-coated beads (GE Healthcare Bio-Sciences AB, Uppsala, Sweden), hybridization of the sequencing primer to the DNA and product washing was done as described previously using a PyroMark^TM^ Vacuum Prep Worktable (Biotage, Uppsala, Sweden) (5). Pyrosequencing was performed at the Genetic Diversity Centre (Zürich, Switzerland) using a PyroMark Q96 ID (Biotage, Uppsala, Sweden) system.

## **Plasmid construction and gene replacement of *L. plantarum* NZ3400B LP_RS14990 gene**

The knockout plasmid was generated *in silico* using a cassette encoding an rRNA adenine N-6-methyltransferase erythromycin resistance gene (*ery*) (6) flanked by the 1050 bp up- and downstream sequence of the LP_RS14990 gene with 50 bp overlap with the LP_RS14990. This sequence was synthesized at Biocat and copied into the pUC18 vector (Biocat, Heidelberg, Germany), resulting in the LP_RS14990 gene replacement vector pUC18_lp_lamC. The vector was transferred into calcium competent *E. coli* MC1000 (7). *E. coli* MC1000 was grown overnight in Brain Heart Infusion Broth (BHI, Labolife Sàrl, Pully, Switzerland) at 37°C with agitation. The vector was then isolated from full-grown cultures using the PureLink® Quick Plasmid Miniprep Kit (Invitrogen, Thermo Fisher Scientific Inc., Waltham, USA). The purified plasmid was transferred to *L. plantarum* NZ3400B by electroporation using an Eppendorf Eporator® (Eppendorf, Hamburg, Germany) as described previously (8). Plasmid integration was detected by plating on MRS agar + 50 µg/ml erythromycin. Double cross-over strains in which LP_RS14990 was replaced by *ery,* were identified by PCR using specific primers (see Table S2 in the supplemental material) targeting the LP_RS14990 gene (lamC-5’, lamC-3’) and primers binding outside the flanking region cloned in pUC18_lp_lamC vector (lamC-D-5’, lamC-U-3’) and the erythromycin gene (ery-5’, ery-3’).

**References**

1. Martin M. Cutadapt removes adapter sequences from high-throughput sequencing reads. EMBnet Journal 2011;**17**:10-2.

2. Callahan BJ, McMurdie PJ, Rosen MJ, Han AW, Johnson AJA, Holmes SP. DADA2: High-resolution sample inference from Illumina amplicon data. Nat Methods 2016;**13**:581-+.

3. Quast C, Pruesse E, Yilmaz P, Gerken J, Schweer T, Yarza P *et al.* The SILVA ribosomal RNA gene database project: improved data processing and web-based tools. Nucleic Acids Res 2013;**41**:D590-D6.

4. Royo JL, Hidalgo M, Ruiz A. Pyrosequencing protocol using a universal biotinylated primer for mutation detection and SNP genotyping. Nat Protoc 2007;**2**:1734-9.

5. Gharizadeh B, Akhras M, Nourizad N, Ghaderi M, Yasuda K, Nyrén P *et al.* Methodological improvements of pyrosequencing technology. J Biotechnol 2006;**124**:504-11.

6. Lambert JM, Bongers RS, Kleerebezem M. Cre-lox-based system for multiple gene deletions and selectable-marker removal in *Lactobacillus plantarum*. Appl Environ Microbiol 2007;**73**:1126-35.

7. Sambrook J, Fritsch EF, Maniatis T. Molecular Cloning: a Laboratory Manual 2nd ed: Cold Spring Harbor, NY: Cold Spring Harbor Laboratory; 1989.

8. Josson K, Scheirlinck T, Michiels F, Platteeuw C, Stanssens P, Joos H *et al.* Characterization of a gram-positive broad-host-range plasmid isolated from *Lactobacillus hilgardii*. Plasmid 1989;**21**:9-20.
